# Supplementary material for: Self-construal and attentional biases in cognitive processing: Insights from Chinese college students for mental health contexts
Source: PLOS Ment Health. 2024 Jun 4;1(1):e0000002. doi: 10.1371/journal.pmen.0000002 (PMC12798562; doi:10.1371/journal.pmen.0000002)
Supplement: Attachment — Submitted filename: Response to Reviewers.docx [file pmen.0000002.s001.docx]

Dear Editors:

Thank you for your letter concerning our manuscript entitled “Self-Construal and Attentional Biases in Cognitive Processing: Insights from Chinese College Students for Mental Health Contexts”(ID: PMEN-D-23-00041). We have made correction in the paper which we hope meet with approval.

**Response to Editor:**

- Please include a separate legend for each figure in your manuscript.

**Response:** we have included a separate legend for each figure in our manuscript.

**Response to Reviewer #1:**

-The manuscript contains bullets and numbers used unnecessarily. These must be removed and prepare the document adequately. title is written well. Data analyses are good. Study seems to be significant but needs minor revision before publication.

**Response:** We have carefully reviewed the entire manuscript, particularly the sections you pointed out as potentially problematic, to ensure that all information is presented in a coherent and logical paragraph format, eliminating the unnecessary reliance on bullet points and numbers. Additionally, we are deeply grateful for your positive remarks about the manuscript’s title and the data analysis. We also concur with your view on the significance of our research and have made minor revisions to the manuscript in accordance with your suggestions, to ensure its quality is optimal for publication.

**Response to Reviewer #2:**

Thank you for submitting your manuscript titled “Self-Construal and Attentional Biases in Cognitive Processing: Insights from Chinese College Students for Mental Health Contexts” for review. Your work contributes significantly to cultural psychology by elucidating the relationship between self-construal and cognitive biases. However, there are several methodological and theoretical aspects that warrant further attention and clarification.

**Methodological Considerations:**

The study discusses the link between self-construal, cognitive biases, and mental health, yet it does not include direct measures of anxiety and depression. While the use of Rosenberg’s Self-Esteem Scale highlights the role of self-esteem in mental health outcomes, incorporating direct assessments of anxiety and depression would substantively strengthen the implications of your findings in the context of mental health.

Additionally, the manuscript omits details about whether participants were screened for other mental health pathologies that could affect their responses to the questionnaires. Including such screening processes would enhance the validity of your findings, ensuring that the observed cognitive biases are more directly related to self-construal and not influenced by other mental health conditions.

Your employment of Derryberry and Reed’s Attentional Control Scale for assessing attentional control is commendable. However, the study lacks a comprehensive cognitive or executive function screening. Given the potential impact of these functions on cognitive biases and mental health, the inclusion of additional cognitive assessments could provide a more holistic understanding of the underlying mechanisms. A sole reliance on one scale might not adequately analyze attentional control in its entirety.

The study’s dependence on self-reported measures could introduce biases such as social desirability. These limitations should be acknowledged and discussed in the manuscript to provide a more balanced view of the results.

**Discussion:**

Grounded in Markus and Kitayama’s self-construal theory, your study provides important insights. However, exploring additional theoretical frameworks could further illuminate the complex dynamics between cognitive biases and mental health. This approach would offer a broader perspective and deepen the understanding of these relationships.

**--Response:** Thank you for your insightful and constructive feedback on our manuscript. We appreciate your recognition of our contribution to cultural psychology and agree that the methodological and theoretical aspects you have raised are crucial for the robustness of our study. Below, we outline our responses to each of your points:

**1. Incorporating Measures of Anxiety and Depression:**

Thank you very much for your suggestion to incorporate direct measures of anxiety and depression into our study to strengthen its implications in the context of mental health. We fully agree that such measurements would enhance the depth and breadth of our research.

However, we are faced with a practical challenge. The data collection for this study was completed in November 2021, involving 748 students. Due to the time lapse and practical constraints, it is not feasible for us to conduct additional tests for anxiety and depression levels on the same cohort of students. Conducting these tests now would not allow us to effectively compare the newly collected data with the previous data, as the time points differ.

We recognize this as a limitation of our study and clearly state this in the revised manuscript. Nonetheless, we believe that even with these limitations, our research still offers valuable insights for college students’ mental health education and psychological interventions. Future research could build on our work to further explore the direct measurement of anxiety and depression levels, thereby enhancing understanding of these complex relationships.

**2. Screening for Other Mental Health Pathologies:**

Response: Your point about screening participants for other mental health pathologies is well-taken.

Action: We have revised the methodology section to include details about our screening process for other mental health conditions. This will ensure the validity of our findings, confirming that the observed cognitive biases are primarily related to self-construal.

**3. Comprehensive Cognitive or Executive Function Screening:**

Thank you for your suggestion to conduct a more comprehensive cognitive or executive function screening. We understand that such assessments could add deeper insights to our study, especially in analyzing attentional control.

However, it is important to note that since the data for this study was collected in November 2021, involving 748 students, it is not feasible to add additional cognitive tests, such as the Stroop Test or the Wisconsin Card Sorting Test, within the current research framework. These tests would need to be conducted simultaneously with the other data collection to ensure consistency and comparability of results. We recognized that this is a limitation of our study design and clearly stated this in our manuscript.

**4. Limitations of Self-Reported Measures:**

We agree that the reliance on self-reported measures can introduce biases like social desirability and include a section in the manuscript discussing these limitations and their potential impact on the study’s results, thereby providing a more balanced view.

**5. Exploring Additional Theoretical Frameworks:**

Response: We value your suggestion to explore additional theoretical frameworks beyond Markus and Kitayama’s self-construal theory.

Action: In the revised manuscript, we expanded our background and discussion to include the Biopsychosocial Model, to deepen the understanding of the interplay between cognitive biases and mental health.

We hope these modifications and clarifications will address your concerns and enhance the overall quality and impact of our research. Thank you again for your thorough review and valuable suggestions.
